# The Reported Few Cases and Deaths of Covid-19 Epidemic in Africa Are Still Data Too Questionable to Reassure About the Future of This Continent

**DOI:** 10.3389/fpubh.2021.613484

**Published:** 2021-02-05

**Authors:** Maria Grazia Dente, Carlo Vittorio Resti, Silvia Declich, Giovanni Putoto

**Affiliations:** ^1^National Center for Global Health, Istituto Superiore di Sanità, Rome, Italy; ^2^Public Relations Office, San Camillo-Forlanini Hospital, Rome, Italy; ^3^Planning and Operational Research Department, Doctors With Africa Collegio Aspiranti e Medici Missionari (CUAMM), Padova, Italy

**Keywords:** COVID-19, Africa, surveillance data, containment measures, health service access and utilization

## Introduction

More than 10 months after the first case of COVID-19 in Africa was detected (in Egypt on February 14), prevalence and mortality are still relatively low and, although there are many hypotheses, the reasons remain unclear (1–3).

Reduced virulence of SARS-CoV-2 in Africa, genetic or trained immunity, and young population (3, 4) are among the main reasons being evaluated.

## Reliability of the Data for Hypothesis and Conclusions

However, it should be considered that conclusions based on the limited available data might be misleading, also considering the fragility of the surveillance and health systems in many African countries and the possible weakness of their data. In addition, data governance in emergency contexts in Africa is historically difficult and some evidence shows that several African countries exert a tight control on public data and information (3, 5), particularly in the case of epidemics.

The WHO African Region reports that complete data on age and gender distribution are presently available only for around 1% of total confirmed cases and that the recent observed decline of cases should be interpreted with caution as many factors could explain this trend, including, but not limited to, changes in testing capacity and strategy, and reporting delays (6). In fact, low numbers of performed tests and high variability within national testing strategies (i.e., due to scarce resources, some countries are testing only symptomatic cases) do not allow monitoring the actual entity of the pandemic in the countries.

Although it increased since the starting of the pandemic, the total tests per population is still <10 per 1,000 in many countries. On December 7, the total daily COVID-19 tests in Africa oscillates between 0 and 1/1000 for around 20 countries with available data (7).

With limited testing, the positive rate might give some suggestions on the progression of the epidemic. Where the number of confirmed cases is high relative to the extent of testing, probably not enough tests are being carried out to properly monitor the outbreak. In such countries, the true number of infections may be far higher than the number of confirmed cases. “And where the positive rate is rising in a country, this can suggest the virus is actually spreading faster than the growth seen in confirmed cases” (7).

Less than 5% of samples positive for COVID-19, at least for the last 2 weeks (assuming that surveillance for suspected cases is comprehensive), is one of the WHO criteria that indicate that the epidemic is under control (8).

In those African countries whose data are available, the positive rate on December 8 oscillates between 2.2 (Togo) and 28.2 (Democratic Republic of Congo), with increasing trend in some cases. However, how these values can be interpreted when very low number of tests are performed (Togo 0.11/1000 and DRC <0.01/1000)? (7).

In countries where data are collected, indications on the entity of this pandemic are provided by the excess of mortality, as reported by the South African Medical Research Council. From 6 May to 14 July, South Africa reported 4.453 COVID-19 deaths, but experienced 17.090 more deaths from all natural causes than would be expected based on historical averages. These excess deaths might be attributed to unreported COVID-19 deaths as well as due to other diseases, as health services are re-orientated, but South African researchers deem that COVID-19 underreporting has the biggest role in these unexplained excess deaths (9).

It is also challenging to understand what worked and what did not without reliable data that allow comparison (10). There has been limited testing of asymptomatic cases or of antibody titers to evaluate successes of early interventions in preventing transmission or possible differences in susceptibility between populations of different regions (3, 11). It is also plausible that not all countries have been able to implement and maintain the same containment and control measures for the epidemic over time. Therefore, comparisons are risky, and it is difficult to identify “winning and effective” strategies without considering specific national situations (12–15) as in the recent attempt done in Mali, Burkina Faso, Senegal, and Guinea (16). Often, as in these four countries, the strategies adopted are very similar to those applied in high-income countries, but the contexts are very different, and this seems to lead to suboptimal results. This is the case of measures that have had a strong impact on the existing social and cultural realities, compromising their acceptability within the communities.

In Nigeria, a multisectoral approach was planned including various levels of lockdowns and ban of gatherings. However, the envisaged mobilization of relevant stakeholders was partially missing, faith leaders were not appropriately engaged, and, despite the ban, they conducted congregation services (17); in Ghana, lockdown measures were activated from the earliest cases, but the epidemic was significant even if with a reported low case fatality ratio (0.6%) (6, 18).

## Impact of COVID-19 Epidemic's Containment Strategies and Measures

On the basis of what has been reported and discussed in the previous section, the relatively low number of cases and deaths of COVID-19 reported in some African countries might be really far from the real situation. Moreover, the overall impact of the containment strategies and measures should not be underestimated.

Worldwide, lockdown and containment measures have posed major challenges, and the restrictive provisions needed to detect, test, isolate, and track positive cases of SARS-CoV-2 infection involve a very broad spectrum of activity and deeply affect national socio-economic dynamics.

The need for considerations on the impact of these measures is therefore fundamental and even more stringent as regards fragile states. For example, in order to flatten the outbreak curve, some African governments have imposed severe public health measures based on physical distancing to reduce transmission. However, the repercussions of this approach in poor communities may have been underestimated, and it is plausible that, ultimately, the lives lost due to the lockdown could outweigh those saved by COVID-19. In fact, some unwanted and potentially fatal consequences of social isolation are threatening the livelihood of African citizens, worsening the economic situation and increasing food insecurity, finally affecting also social stability and the genuine efforts of some countries in transition toward possible horizons of democracy (16, 19–23).

There is a need for targeted containment interventions monitored over time based on context-specific evidences that gradually consolidate. For example, outcomes from response to the COVID-19 epidemic in Zimbabwe suggest the restriction of the movement of people between different suburbs and between urban and rural areas while allowing some level of economic activity in association with active surveillance and testing for both imported and community cases (24, 25).

Some recent modeling studies show that before implementing travel restrictions, local COVID-19 incidence, local epidemic growth, and travel volumes should be considered, as restrictions seem to affect epidemic dynamics only in countries with low COVID-19 incidence and large numbers of arrivals from other countries, or where epidemics are at exponential growth (26).

The WHO “Pulse survey on continuity of essential health services during the COVID-19 pandemic” reported that all services were affected, including essential services, in nearly all countries, and more so in lower-income than higher-income countries (27, 28).

In many African countries, COVID-19 is “among” the country's epidemics, along with cholera, measles, malaria, and Ebola (29, 30). The COVID-19 epidemic response has exacerbated these fragile situations by reducing access and service delivery, as consequences of re-orientation of services, reduction of mobility, and fear of using the services. The focus on COVID-19 has diverted the attention away from the common pediatric infectious diseases, reproductive health care, the management of obstetric complications, and the provision of routine immunization services, which has been substantially blocked in at least 68 countries around the world, putting around 80 million children under the age of 1 at risk (31, 32).

Modeling estimations show that if the COVID-19 pandemic results in widespread disruption to health systems, childbirth care and child curative services will be the most affected and would account for the greatest number of additional maternal and child deaths (33).

Large numbers of patients in Africa with HIV and tuberculosis are dependent on functional health services, and if access to treatment is reduced or interrupted, the consequences for individual and public health can be substantial (34, 35).

The phenomenon is well-known, especially in Africa. The results from the modeling estimations done after the West African Ebola outbreak of 2014 are emblematic. They showed how dramatic was the impact on malaria, HIV/AIDS, and tuberculosis mortality rates through reduced access to treatment for varying reductions in treatment coverage. The modeling study indicated that 11,300 deaths from the Ebola virus had been nearly matched by 10,600 excess deaths from other diseases, especially malaria, HIV/AIDS, and tuberculosis (9, 36).

Fortunately, there is now awareness on these critical unwanted consequences, and efforts are ongoing to identify viable solutions (37). At this point, the hope is that health systems will not disrupt, but rather strengthen themselves to face the imminent challenges posed by COVID-19 vaccinations (38).

## Discussion

The monitoring of the progression of the COVID-19 pandemic in Africa is presently very challenging, mainly due to the very fragile health systems involved, which are struggling to implement containment strategies and to collect data to monitor the situation.

However, even if over time we had more data to monitor the pandemic in the continent, the overall consequences are easily predictable now, even without further modeling studies.

In this critical situation, the health workforce has also decreased, considering that, as per september 2020, more than 42,000 health workers were infected in Africa since the start of the pandemic with around 880 deaths (2.1% CFR) (6).

In addition, the deterioration of the global economy because of the COVID-19 pandemic predicts an increase in absolute poverty (39, 40) and compromises the achievements of the Sustainable Development Goals (41) with additional harsh repercussions on the African continent.

In this situation, an unprecedented and participatory effort involving all stakeholders is requested to identify strategies that can contain the extent of this current protracted emergency without affecting the delivery of primary health care services. The African continent cannot afford the further weakening of its already fragile health system, and the tightness of the system will both increase the availability of reliable data and help to cope with the other dire consequences of this epidemic. Now, more than ever, effective aid and sound cooperation are to be sustained.

## Author Contributions

MGD drafted the first version of the article. CVR, SD, and GP revised and integrated it. All authors contributed to the article and approved the submitted version.

## Conflict of Interest

The authors declare that the research was conducted in the absence of any commercial or financial relationships that could be construed as a potential conflict of interest.
